# Users’ Perceptions Toward mHealth Technologies for Health and Well-being Monitoring in Pregnancy Care: Qualitative Interview Study

**DOI:** 10.2196/28628

**Published:** 2021-12-02

**Authors:** Jane Li, David Silvera-Tawil, Marlien Varnfield, M Sazzad Hussain, Vanitha Math

**Affiliations:** 1 Australian e-Health Research Centre Commonwealth Scientific and Industrial Research Organisation Marsfield Australia; 2 Australian e-Health Research Centre Commonwealth Scientific and Industrial Research Organisation Herston Australia; 3 Department of Obstetrics and Gynaecology Gold Coast University Hospital Gold Coast Australia

**Keywords:** pregnancy care, wearable sensors, mobile health, acceptance, mHealth service, design, mobile phone

## Abstract

**Background:**

Mobile health (mHealth) technologies, such as wearable sensors, smart health devices, and mobile apps, that are capable of supporting pregnancy care are emerging. Although mHealth could be used to facilitate the tracking of health changes during pregnancy, challenges remain in data collection compliance and technology engagement among pregnant women. Understanding the interests, preferences, and requirements of pregnant women and those of clinicians is needed when designing and introducing mHealth solutions for supporting pregnant women’s monitoring of health and risk factors throughout their pregnancy journey.

**Objective:**

This study aims to understand clinicians’ and pregnant women’s perceptions on the potential use of mHealth, including factors that may influence their engagement with mHealth technologies and the implications for technology design and implementation.

**Methods:**

A qualitative study using semistructured interviews was conducted with 4 pregnant women, 4 postnatal women, and 13 clinicians working in perinatal care.

**Results:**

Clinicians perceived the potential benefit of mHealth in supporting different levels of health and well-being monitoring, risk assessment, and care provision in pregnancy care. Most pregnant and postnatal female participants were open to the use of wearables and health monitoring devices and were more likely to use these technologies if they knew that clinicians were monitoring their data. Although it was acknowledged that some pregnancy-related medical conditions are suitable for an mHealth model of remote monitoring, the clinical and technical challenges in the introduction of mHealth for pregnancy care were also identified. Incorporating appropriate health and well-being measures, intelligently detecting any abnormalities, and providing tailored information for pregnant women were the critical aspects, whereas usability and data privacy were among the main concerns of the participants. Moreover, this study highlighted the challenges of engaging pregnant women in longitudinal mHealth monitoring, the additional work required for clinicians to monitor the data, and the need for an evidence-based technical solution.

**Conclusions:**

Clinical, technical, and practical factors associated with the use of mHealth to monitor health and well-being in pregnant women need to be considered during the design and feasibility evaluation stages. Technical solutions and appropriate strategies for motivating pregnant women are critical to supporting their long-term data collection compliance and engagement with mHealth technology during pregnancy.

## Introduction

### Background

Pregnancy is a normal physiological process, with most pregnancies progressing without any problems. However, pregnancy may pose many risks and complications (eg, gestational diabetes mellitus [GDM], preeclampsia, and mental health problems), which might greatly affect the health of the mother, fetus, or both [1,2]. Together with women’s existing medical conditions (eg, diabetes and hypertension), pregnancy-related complications can lead to adverse outcomes such as the loss of pregnancy by miscarriage, stillbirth, or low birth weight [1,2]. A healthy lifestyle is essential for the health of the mother and fetus and can potentially reduce the risk of maternal complications [3,4].

The pregnancy journey involves regular checkups that allow clinicians to monitor progress, identify potential risks, and provide general advice to encourage a healthy lifestyle [5]. Different factors may contribute to the likelihood of developing pregnancy-related conditions. Individual risk factors (eg, age and BMI), lifestyle patterns (eg, diet and physical activity), and physiological measures (eg, blood glucose levels, blood pressure, and proteinuria) are all indicators of pregnancy-related risk conditions [6,7]. Regular health and well-being monitoring can support early detection of health risks, improve treatment, and promote lifestyle adaptions in pregnant women [8,9].

Mobile health (mHealth), which involves the use of mobile and wireless technologies to support the achievement of health objectives [10], has been widely used in health care [11-13]. Wearable sensors and health monitoring devices are becoming popular and being used to support the monitoring of health and well-being [12-15]. These technologies support the sensing, tracking, and reporting of individuals’ health measures continuously (eg, physical activities and physiological data). Smartphone apps, coupled with wearable and sensing devices, have been used as data interfaces for visualization of measurement data, as motivational tools via persuasive messages, and to support personalized digital interventions to improve care programs [16,17]. With the availability of these technologies in health care, passive monitoring and personalized assessment would become integral to continuous patient monitoring [18].

The role of mHealth during pregnancy is being increasingly investigated [19-23]. Using apps to support pregnant women enhances the traditional pregnancy care model by providing additional educational information and empowering women to look after their own health [21,23-26]. Sensors and monitoring technologies that automatically track specific health indicators have been integrated into mHealth solutions to support pregnant women’s self-care behaviors [22,27-29].

### Challenges in Pregnancy mHealth Care

Despite the expansion of mHealth, the practicality, design, and user needs for digital health monitoring in pregnancy require more attention [23,30]. There is a range of consumer-based wearable sensors and prototypes that can measure the physical activity, sleep, and physiological parameters [18,31,32]. However, none of these have been specifically designed for pregnancy care. While research has explored women’s and clinicians’ views of mHealth in pregnancy, there is still a knowledge gap regarding the preferences of mHealth monitoring among pregnant women and their clinicians as well as the suitability of mHealth monitoring for different conditions [24,29,30,33]. Recent studies have highlighted the importance of patient-centered design and behavior decision research in the development of mHealth solutions for pregnancy [19,23,28]. Understanding the women’s and clinicians’ preferences and their existing and preferred monitoring practices is crucial to assist in the design of practical solutions to promote a healthy lifestyle during pregnancy [23,24,28].

Intelligent data analysis can be used to identify the early signs of illness [34] and potentially support the early detection and management of complications in pregnancy [35,36]. Previous studies have investigated the use of predictive analytics and apps to support pregnancy care, with a focus on specific conditions and the medical data collected by clinicians [8,37]. These solutions required access to data in medical record systems and did not consider lifestyle (eg, sleep, diet, and exercise) factors in their analysis. The ability to extend the capability of clinical monitoring with multidimensional health and well-being data, collected via wearable and health monitoring devices, has the potential to provide significant benefits to the pregnancy journey. However, challenges remain in the collection of large-scale and long-term quality data deemed suitable for pregnancy care [19,23].

Related to these challenges is the need to support pregnant women’s engagement with technology [24,25,27,28,30]. Even with emerging evidence on the potential benefits, barriers to the implementation of mHealth technologies in pregnancy care persist [19,24,27,28,38]. Various factors can impact an individual’s feeling toward sharing and tracking health data, including stress associated with mHealth monitoring, the availability of reliable educational information, and ineffective communication with clinicians [22,25,27,30]. Further research is needed to understand pregnant women’s motivation to use technologies to better support their engagement, data collection compliance, and daily use [27,33]. This understanding can inform the development of mHealth lifestyle interventions and the integration of mHealth into pregnant women’s daily routine and the clinicians’ care practices [24,25,30,38].

### Objective

As an initial step to inform the design of appropriate technologies to support the monitoring of health and well-being during pregnancy, we aim to conduct a qualitative study with clinicians, pregnant women, and postpartum women to understand their existing risk assessment and monitoring practices as well as their needs, interests, and preferences in mHealth. We also aim to explore the potential factors that may influence their engagement with mHealth data collection and monitoring as part of pregnancy care. The study, conducted in the Australian context, contributes to the broader understanding of factors that motivate the use of mHealth in pregnancy care and how novel technologies can be designed and introduced to improve user engagement and long-term health monitoring during pregnancy.

## Methods

### Overview

A qualitative study was conducted using semistructured interviews with pregnant women, postpartum women, and clinicians currently working in pre- and postnatal care. The study was approved by the Commonwealth Scientific and Industrial Research Organization Health and Medical Human Research Ethics Committee (reference number: 2019_017_HREC) and the Gold Coast Hospital and Health Service Human Research and Ethics Committee (reference number: LNR/2019/QGC/54173) in Australia.

The interview questions were adapted from previous studies in conducting qualitative studies on digital health technology design [39,40]. The interviews with pregnant women and postpartum women included questions related to their experience in monitoring their health and well-being during their pregnancy, in particular their previous and current use of digital health technology (activity trackers and health monitoring devices and apps), and their interest and intention to use technology to support the management of their pregnancy needs. The motivations and factors that would contribute to their potential acceptance of mHealth monitoring were also explored. In addition, basic demographic and general information about their lifestyle during pregnancy was collected.

The clinician interviews included questions related to pregnancy risk assessment and management as well as supporting pregnancy health and well-being in standard clinical care. Clinicians’ thoughts were also collected on monitoring the components of mHealth solutions, on potential medical conditions for mHealth monitoring, and on how to introduce mHealth monitoring in pregnancy care. Basic demographic information about their roles and years of experience was also collected.

### Participants

#### Female Participants

The criteria for pregnant and postpartum female participants (from here on, termed *female participants*) included the following: an age of ≥18 years, pregnancy (any stage of gestation) or postpartum pregnancy (no longer than 6 months postbirth) at the time of study, and the ability to give informed consent. The female participants were initially recruited via 2 internal email mailing lists within the authors’ organization. The email recipients were asked to share the invitation with friends and relatives who might be interested in participating. A snowballing technique was also used, asking women who had been interviewed to suggest other potential participants. Once an expression of interest was received, an information sheet and a consent form were sent to the potential participants via email, and interviews were scheduled after the participants consented. Purposive sampling was used during recruitment, with participants selected by considering their pregnancy stages. The focus was on a typical pregnancy, and high-risk pregnancies were not targeted. Initial data coding was performed during the data collection process. Recruitment continued until no new codes were identifiable in the subsequent interviews. The female participants were offered a gift voucher value of Aus $60 (US $42) as compensation for their time.

#### Clinician Participants

Clinicians recruited for the interviews were health care professionals involved in pre- and postnatal care at the obstetrics and gynecology department of a tertiary public hospital in Australia, which delivers standard clinical services in pre- and postnatal care. The management team of the department was interested in exploring the use of mHealth technology. Potential participants were identified by a key study representative (an obstetrician) at the hospital. They were chosen based on their role, level of experience, and interest in participation. The study representative emailed potential participants the information sheet and consent form to introduce the study and study investigators. Clinicians were required to express their interest in participating before being included in an interview. No compensation was offered to the clinician participants, except for a chocolate snack after the interview.

### Study Procedure

All interviews were conducted on a one-on-one basis with each female participant during a 3-month study period. They were all offered the choices of face-to-face, videoconference, and telephone interviews. To ensure that the clinician participants were less inconvenienced, 2 researchers made themselves available at the hospital for 2 full days. Clinician participants could attend the interviews anytime during those 2 days, if their workflows allowed. Face-to-face interviews were conducted either at a clinician’s private office or at a small meeting room in the hospital. In addition, a telephone interview was offered to a clinician who visited a different hospital. The interviews were scheduled with the help of the study representatives. Each interview session was conducted with 1 clinician, except for 1 session involving 2 nurse managers. Each interview was conducted by 2 researchers and lasted approximately 30-45 minutes. The interviews were audio-recorded and transcribed professionally.

### Data Analysis

A thematic coding technique was used to identify the insights from the interview data. One key researcher worked on the coding of the transcripts using NVivo (QSR International) data analysis software, and the other 2 researchers summarized their interview notes. Initial findings regarding the themes that emerged were discussed among the research team members. A second round of coding and analysis was conducted by a key researcher. The discussion continued over several meetings before a report of the findings was generated. Themes related to the current practices of regular monitoring and risk assessment, current experiences, and motivations for using mHealth technologies, and perceived benefits and challenges in incorporating mHealth technologies in daily life and practices were identified.

## Results

### Participant Characteristics

A total of 8 female participants were interviewed (Table 1); 2 (25%) of them were in the second trimester of pregnancy, 2 (25%) were in the third trimester, and 4 (50%) were in the postpartum stage. Pregnant women in their first trimester of pregnancy were not available during recruitment. Of the 8 female participants, 2 (25%) had GDM, 1 (13%) had preeclampsia, 1 (13%) had iron deficiency, and 4 (50%) had no medical conditions during their pregnancies. Moreover, 4 (50%) of the 8 female participants used private hospitals, 3 (38%) used public hospitals, and 1 (13%) had both public and private hospital experiences in their current and previous pregnancies. A total of 4 (50%) female participants were recruited from the researchers’ organization. All (8/8, 100%) of the female participants had experience using smartphones and mobile apps in general. A total of 13 clinicians from the public hospital were interviewed, including 2 (15%) obstetricians who also worked at private hospitals, 7 (54%) midwives in different roles, 1 (8%) health educator, 1 (8%) social worker, 1 (8%) physiotherapist, and 1 (8%) dietitian (Table 2).

**Table 1:** Characteristics of female participants (N=8).

Characteristics			Values, n (%)
**Pregnancy stage**			
	Second trimester	2 (25)	
	Third trimester	2 (25)	
	Postpartum (2-4 months)	4 (50)	
**Medical condition during pregnancy**			
	Gestational diabetes	2 (25)	
	Preeclampsia	1 (12)	
	Iron deficiency	1 (12)	
	No medical conditions	4 (50)	
**Working time during pregnancy**			
	Full-time	7 (88)	
	Part-time	1 (12)	
**First time mother**			
	Yes	7 (88)	
	No	1 (12)	
**Public or private service used**			
	Public	3 (38)	
	Private	4 (50)	
	Mixed	1 (12)	
**Have used smartphones and apps**			
	Yes	8 (100)	
	No	0 (0)	

**Table 2:** Characteristics of clinician participants (N=13).

Characteristics			Values, n (%)
**Roles**			
	Obstetricians	2 (15)	
	Midwife	3 (23)	
	Midwife, manager	2 (15)	
	Midwife, clinical consultant	1 (8)	
	Midwife, general practitioner liaison	1 (8)	
	Health educator	1 (8)	
	Social worker	1 (8)	
	Physiotherapist	1 (8)	
	Dietitian	1 (8)	
**Experience in their fields (years)**			
	<10	2 (15)	
	10-20	6 (46)	
	20-30	4 (31)	
	>30	1 (8)	
**Gender**			
	Female	11 (85)	
	Male	2 (15)	

In this section, we have presented the key themes extracted from the data, which are grouped into the following categories: risk assessment, health monitoring practices, and care needs; female participants’ experience of health monitoring and attitude toward mHealth; and the clinician participants’ perception of mHealth.

### Risk Assessment and Monitoring During Pregnancy

Clinicians pointed out that every pregnant woman has unique care needs and potential risks. They articulated when and how risk assessment and support of lifestyle adaptions could be carried out in practice.

#### Risk Assessment and Care Needs

The need for longitudinal monitoring was highlighted by clinicians because many women who were initially believed to have low-risk pregnancies due to their medical history could become high-risk as their pregnancies progressed; one of the clinician participants (C9) commented “pregnancy is a journey with unpredictability.” As the clinicians explained, specific indications and risks for medical conditions were assessed during the early stages of pregnancy, throughout the pregnancy period, during labor, and during postpartum. In the early stages of pregnancy, the women’s health is typically monitored by their primary care provider, the general practitioner (GP), before their first visit to a hospital or an obstetrician. On the initial visit to a hospital (for women using the public system), medical data from the GP referral, medical history (eg, previous obstetric, family, and psychosocial histories), pre-existing disorders, and lifestyle (eg, smoking and alcohol intake) were consolidated. For women who used private hospitals, obstetricians managed their care and monitoring from the early stages of pregnancy until postpartum. In public hospitals, midwives used the guidelines of referral and consultation to decide how and when to refer women at different stages of pregnancy to obstetricians and other health care professionals. The care plan and monitoring arrangements depended on the women’s condition, their risk factors, their preferences, and the hospital’s capability. One obstetrician explained:

If anything changes along that timescale, they get referred back to obstetric clinic and they come under obstetric care, but that is because they developed a complication along the lines, either would be gestational diabetes, or they have problems with blood pressure or baby is not growing as well as it should be.

C11

Assessment and care coordination is required for high-risk pregnancies. At the public hospital where the study was conducted, a clinical midwifery consultant worked as a navigator to provide coordination and consultation in collaboration with the obstetricians and other specialists for high-risk pregnant women. Such cases were flagged in the electronic medical records and their management plans were recorded. The high-risk pregnant women need to visit the hospital multiple times to see different specialists, whereas others (intermediate risk) whose conditions are well-managed only need some level of coordination. The changing nature and different care needs for pregnant women with different risk profiles are described as follows:

It really depends on the health and well-being of the women, and sometimes I can downgrade women and sometimes I have to upgrade them as their pregnancy goes along. So sometimes, women at the beginning need quite a lot of intensive support and coordination, but once we have got that underway and we are on the right track, then it can be stepped down.

C13

#### Supporting Pregnant Women’s Healthy Lifestyle and Well-being

Clinicians pointed out that awareness and support of physical activity, diet, and mental and emotional aspects of well-being in pregnant women is an important component of pregnancy care. Nutrition, exercise needs, and expected weight gain were discussed with the pregnant women during their first visit. This information was included in the information pack, together with useful website information and web links. Although the low-risk women received general education, specialist consultations were provided for at-risk women (eg, those with diabetes) to guide their diet and physical exercise. Physiotherapy and dietitian support were provided for women who needed interventions. Mental health was assessed by an obstetrician review or a midwife review. The Edinburgh questionnaire [41] was used as a screening tool. Triage assessment for women with high scores was performed, followed by a referral to the mental health team for their ongoing management during the antenatal and postnatal periods.

The midwife manager participants pointed out that there has been some attention to monitor women’s lifestyle behavior, emotional health, and well-being from a longitudinal perspective. There is a trend of expanding the timeline of pregnancy and perinatal care to the first 1000 days from conception to when children turn 2 years old [42]. This is a critical period as the pregnant and postnatal mothers’ health, nutrition, and stress levels can have a long-term impact:

The education around that for the mother...is modifiable behaviours...giving them the right support and the right education that can make a big difference, even the small changes...for the length of stay, and for the long-term health benefits of that child if they can change those behaviours when they go home as well.

C7

### Female Participants’ Perception on Health Monitoring and mHealth

#### Overview

All female participants believed that maintaining a healthy lifestyle was beneficial for their pregnancy. Most of them integrated a certain level of exercise into their daily routines. Walking was the most popular type of activity among them, followed by activities such as swimming and aerobics. They also maintained a balanced diet and monitored their body weight.

In this section, we have described the female participants’ views on health measurements (eg, blood pressure and blood glucose levels) and lifestyle behaviors (eg, physical activity, diet, sleep, stress and mental health, and weight management) that can be tracked using wearable sensor devices, health monitoring devices, and mobile apps.

#### Monitoring Physical Activity, Sleep, and Heart Rate

Commercial activity trackers (eg, Fitbit [Fitbit Inc] and Apple Watch [Apple Inc]) were used by 63% (5/8) of the female participants to track their steps, sleep, and heart rate. Of the 5 of them, 2 (40%) used activity trackers before pregnancy and continued using them during pregnancy, whereas the other 3 (60%) bought a device specifically to monitor their physical activity during pregnancy. Participants who did not own or use activity trackers during pregnancy thought that they had no medical problems, were physically active already and did not need additional motivation, or were concerned about the inconvenience of wearing a tracker and the need to charge the device battery.

Physical activity was the key measure tracked by the participants who used trackers. Furthermore, 25% (2/8) of participants used trackers to keep track of sleep quality. Interestingly, although 4 (50%) of our 8 participants mentioned sleep problems during pregnancy (eg, waking up a couple of times at night and difficulty going back to sleep), they did not feel the need to track their sleep every day as they knew they had this issue. Similarly, heart rate was not a concern for most of them, with only 25% (2/8) of participants who tracked their heart rates on the activity trackers noticing an increased heart rate when they were stressed:

I tended to notice that it seemed to be when I was trying to rush around somewhere or I was a little bit anxious, it was often higher when I was at the hospital...so I was just conscious to kind of take some deep breaths and just try and sit down and calm down for a little bit.

F2

#### Monitoring Weight

All female participants used weight scales at home before and during their pregnancy. Of the 8 participants, 2 (25%) paid particular attention to weight increase as suggested by doctors, whereas another 6 (75%) participants used the scales on a *now and then* basis:

I didn’t properly track my weight, I just put myself on the scale every week or so...And the obstetrician always got my weight as well on his scales, so he tracked it that way.

F5

#### Monitoring Diet

Participants with GDM diagnosis tracked their calories. Of the 8 participants, 1 (25%) tracked food intake using an app and found it useful, as she was told by her physician not to gain more than a certain weight. Half (4/8, 50%) of the participants expressed their willingness to try a diet-tracking app. The other half felt that they did not have any major health concerns that required them to track food intake, that their weight was in healthy range, or that they were concerned about the time and effort required to record and check the data:

That’s never been a priority for me to monitor how many calories I’ve had during the day because I’ve never been someone that over eats and I’ve always stayed within a pretty healthy weight range.

F3

#### Monitoring Blood Pressure

Most of the female participants did not measure their blood pressure at home, with it being measured only during their hospital visits. Only 38% (3/8) of them had a blood pressure device at home, with only 25% (1/4) of them (who used a private hospital) measuring it regularly at home owing to abnormal blood pressure being detected occasionally on her visits to the clinic. Participants were educated by their clinicians on how to monitor their symptoms and were informed that their local GPs or pharmacists could check their blood pressure if needed:

If I had other symptoms, I felt confident that I could just find that out quite easily and quickly, and I know a lot of pharmacies and things they can just do a quick blood pressure check.

F1

#### Monitoring Blood Glucose Level

Female participants with GDM maintained regular self-measurement and reporting of blood glucose levels during pregnancy. The readings from a blood glucose testing device were recorded by women (in a public hospital) in a booklet and discussed with clinicians on their visits. At private hospitals, this was recorded by women on an Excel (Microsoft, Inc) spreadsheet and emailed to the clinics every 1 or 2 days to be reviewed by the hospital’s endocrine or obstetrics team.

#### Monitoring Mental Health

The difficulty in receiving mental health support during the first trimester was expressed by 63% (5/8) of participants, as they were reluctant to talk about their pregnancy at this stage. Some of them had morning sickness and did not enjoy food or exercise. Although a GP is the primary care provider before their first appointment at the hospital (or obstetric clinic), most women had not established regular GP visits in their first trimester.

In terms of tracking mood, it appeared that 25% (2/8) of female participants who had good support network (family and friends) were less interested in tracking their mood, whereas most participants expressed this need as they either felt stressed in maintaining their work commitments or became very emotional during pregnancy:

Problems that probably didn’t seem like a problem before all of a sudden seem so much worse, that sort of gets you down a bit –that sort of feeling like you emotional and you really want to talk about something and – but then you wake up in the morning and you’re like I don’t know what I was upset about, it’s probably that sort of feeling that I’ve had throughout the pregnancy.

F3

#### Using Mobile Apps

All female participants had experience using various apps that provided pregnancy and postnatal information or assisted in the tracking of fetal movements. The key function they used was to obtain information, such as week-by-week information about their pregnancy progress and the baby’s growth, the symptoms to look out for, nutrition information, and mental health support. Apps for postnatal care were also used by 25% (2/8) of participants to track the feeding, sleep, and growth of their babies. Most participants used the apps to receive information than to enter information:

I actually don’t put much information into it, I use it more just for information like sourcing, but it does allow you to track all your appointments and put all your symptoms in and things like that as well...But just having to enter information every day without kind of getting any information back I don’t know that wouldn’t be so appealing.

F1

#### Female Participants’ Attitude Toward mHealth

Female participants’ overall attitude and concerns toward mHealth included the following:

- They would be motivated to use mHealth if they knew that clinicians could access their mHealth data, and they were willing to share and discuss the data with clinicians at the hospital or clinic visits.
- They would prefer to use devices that featured automatic data capture without the need for manual data entry.
- If the use of monitoring devices (eg, blood pressure and blood glucose level) had the potential to be associated with positive outcomes, they would have had a stronger motivation to use them.
- They would be less motivated to use mHealth if they had no medical conditions or potential risks during pregnancy.
- They were concerned that it could be a source of anxiety if the measurements were slightly out of the normal range, adding to the stress they already had during their pregnancy.
- Half of them believed that most of the apps managed data privacy and security well, whereas the other half were concerned about potential security issues with their data. All of them indicated that if there is an assurance of data security and proper use of the data (such as studies aimed at improving pregnant women’s health), they would be more likely to track lifestyle behaviors and health measures.

### Clinician Participants’ Perception on mHealth

#### Overview

Clinician participants saw the potential of using mHealth technologies for the monitoring of health and well-being in pregnant women, particularly for longitudinal monitoring during pregnancy, as complications could develop in women with or without a risk history. They also acknowledged that it could play an important role in supporting the current practices of risk assessment and care for pregnant women with different risk levels. According to them, for pregnant women classified as high-risk and for those who required additional education and monitoring of their health status, mHealth can be an invaluable tool to improve their compliance.

In this section, we have described the clinicians’ views on technology requirements and suitable conditions for mHealth monitoring and the factors that may help introduce technology in their practice.

#### Broad Requirements

Data collection from multiple sources, incorporating accurate information to pregnant women, clinician portal to access data, providing features for alertness, and ease of use were among the desired features for the clinicians.

##### Health Monitoring Data

Clinicians agreed that some generic health parameters, such as blood glucose and weight, could be measured by pregnant women at home. The dietitian participant mentioned that a wearable continuous glucose monitoring device was provided to patients with type 1 diabetes and this was supported by the Australian government’s continuous glucose monitoring initiative [43].

The clinicians’ opinions on the measurement of blood pressure at home by pregnant women varied. Of the 13 female participants, 3 (23%) of them were supportive, whereas others expressed concerns. In total, 6 participants explained that misinterpretation and inaccurate readings can cause unnecessary anxiety as pregnant women are not trained to measure blood pressure correctly; individuals’ reference ranges might also be slightly different for each woman, and clinicians did not expect low-risk women to take their blood pressure at all times. One clinician participant commented the following:

Because they use electronic blood pressure cuffs at home, they are not trained to use a manual one and the electronic ones may not accurate, they can be a bit off and then that could lead to the woman starting to worry and think-oh my blood pressure is so high...If needed we might get them to go to their GP and get it done more frequently...or monitoring blood pressure by visiting the pharmacy.

C8

##### Lifestyle Data

Clinicians were most receptive to the use of physical activities, diet, and sleep monitoring when considering wearable sensors and apps for pregnant women. One clinician mentioned that some existing blood glucose monitoring apps can collect additional information such as self-reported insulin dose, dietary intake, carbohydrate amounts consumed, and the time of exercise. Another clinician pointed to the link between good sleep and clinical outcomes, such as blood glucose level control. Physical activity tracking was considered particularly useful for women with a high BMI or with diabetes:

Physical activity is helpful for women, but in terms of us it is not something we ask normal women like we won’t say have you been walking three times this week, but it is relevant if she has diabetes where she needs to do the physical exercise of if she has a higher BMI.

C8

##### Questionnaire Data

Incorporating validated questionnaires (such as the Edinburgh questionnaire to measure the risk of mental health issues) into an mHealth solution was suggested by some clinicians. However, they also pointed out that the questionnaire results would need to be analyzed in combination with other measures. The frequency of the questionnaire should be considered on a case-by-case basis, and guidelines were needed for clinicians to follow up on the results, as one clinician explained:

We have to have some process of being able to pick that up and work with, say if someone reports that they’re not doing so well or they have suicidal ideation we have to make sure we have clear pathways of what to do with that information.

C6

##### Incorporating Accurate Information and Feedback

Clinicians saw the importance of incorporating an app providing tailored information and feedback to pregnant women into the mHealth technical solution.

According to the clinicians, pregnancy is a process of education and information seeking for pregnant women. Not all pregnant women read the material provided by hospitals. Some might not know their risks, the consequences of the risks, and the symptoms to watch. They might seek materials from the internet or educational content from the available apps that provide general information. Hospitals did not provide suggestions for the selection of apps.

Clinicians highlighted that educational content needs to be accurate and tailored to particular conditions. Ideally, it should provide individualized information or advice to women with different risk factors and should integrate targeted information as a component to encourage the women’s use of mHealth during pregnancy. One clinician said, “It’s just got to be continually meeting the needs of the different cohorts and the health literacy of the individual” (C7).

##### Access Data by Clinicians

Clinicians discussed the need for a portal or central source of mHealth data for different clinicians to access. The ability to review the data can better support patient-clinician communication and improve the efficiency of face-to-face consultations.

##### Alerts

The clinicians were supportive of an alert feature. They pointed out that one key aim of monitoring should be to make women respond to their data, that is, women getting flagged by an alert that could trigger their access to health professionals. Predictive analysis based on the monitoring data could also potentially provide alerts to clinicians to allow early detection of problems and timely interventions.

##### Ease of Use

Clinicians highlighted that mHealth technology needed to be user-friendly and with minimum effort to use for pregnant women. They suggested that mHealth solutions incorporate monitoring devices with the feature of automatic data capture.

#### Clinical Considerations for mHealth Monitoring

The conditions in pregnant women groups that can potentially benefit from mHealth technology-assisted monitoring were discussed by the clinicians.

##### Gestational Diabetes

Pregnant women diagnosed with GDM receive ongoing support from diabetes educators, diabetes dietitians, and endocrine specialists from the pregnancy to postnatal stages. According to our clinician participants, women with GDM were advised to test their glucose levels at home 4 times a day using a glucose monitor, record the results, and have periodic follow-up visits at the hospital. Existing glucose monitoring practices require extensive effort from both female patients and clinicians. Clinicians could benefit from easy access to data through web-based or mobile solutions. Our clinician participants indicated that GDM is the most common and suitable medical condition to consider for mHealth interventions.

##### Hypertension

For women with hypertension, blood pressure needs to be monitored for potential risks of preeclampsia, or to assess the patient’s response when taking medication for high blood pressure. It is important to provide training to these women on how to use blood pressure monitoring devices. Blood pressure measures also need to be assessed in combination with symptoms such as headache, blurred vision, and swelling as well as pathology tests.

##### Obesity or High BMI

Women with a high BMI might have pre-existing diabetes or are at a high risk for gestational diabetes. It is important for them to maintain an appropriate lifestyle. They can benefit from regular weight tracking and diet monitoring.

##### Mental Health

This includes antenatal and postnatal depression, anxiety, and depression. mHealth has the potential to help track how pregnant women feel, their mood, and when to receive timely intervention and counseling.

##### Stillbirth Prevention

Obstetrician participants pointed out that despite previous efforts to improve care and monitoring, progress in reducing stillbirth rates remains low. Sleep apnea can be a risk factor for stillbirth, and obstetricians were interested in investigating the relationship between sleep abnormalities and stillbirth using smart sensor technologies.

##### Other

Women with previous history of pregnancy problems, such as fetal growth restriction and fetal loss, were discussed by the clinicians.

#### Challenges

Although the clinicians were positive about the potential uses of mHealth technologies, challenges around engaging pregnant women, technology issues, changes in practices, and evidence-based solutions were discussed in the interviews.

##### Women’s Engagement

According to clinician participants, pregnant women’s compliance with self-reporting (such as diet and questionnaires) could be a challenge, especially for women who were busy and if the recording process was not simple. Women might stop using the monitoring devices if monitoring created anxiety. Language and cultural barriers can be an issue for the engagement of non–English-speaking groups. Although pregnant women diagnosed as high-risk tend to be more compliant, providing education and showing benefits can potentially improve data collection compliance for other women:

I would worry about the compliance that was going to be my first feedback...their lives are so busy and their stress levels fluctuate a lot and they would find it hard to commit all the time...I think the compliancy is as good as the education they’re given, if we explain it well and how it can benefit them and empower them...So the biggest blockage for technology is compliance and consistency.

C3

##### Technology

Technology concerns were captured during the interviews. These included accuracy of wearable sensors and devices, as it was directly linked to the reliability of the data, and the cost of a device, as it was an issue for women with low income if the device was expensive. Therefore, providing individualized information was a challenge, as it would be difficult to meet the complex needs of different women and their different conditions.

Intelligent modeling for prediction and its accuracy can be challenging. From a medical perspective, predicting a medical condition is not easy, according to one obstetrician:

At this stage, in terms of finding predictors, in the first trimester, second, early second trimester, that would predict things like preeclampsia, things like gestational diabetes, things like growth restriction or foetal demise, even now they're still not there, they don’t exist.

C11

An obstetrician pointed out that detection of clinical abnormalities can be enhanced by including other clinical and health data from electronic health record systems. However, the integration with other record systems can be a challenge.

##### Change of Practices

Few self-reported measures (except for blood glucose level for patients with diabetes and weight tracking at some hospitals) were collected in standard practice at the time of our study. The midwives did not collect objective physical activity data and diet information from low-risk women.

Reviewing the women’s monitoring data when introducing mHealth was raised as a concern by some clinician participants (eg, obstetricians) as it would require extra work in their already busy schedules. However, other clinicians (eg, midwives) responded that this would not be an issue for them, but it might need a dedicated staff member to take the responsibility and time to check the data and follow up when needed. Intelligent decision-making with alerts for abnormal measures was considered helpful for clinicians.

There is also a need to improve communication between clinicians from multiple disciplines, including GPs. Engaging busy clinicians by showing potential outcome improvements could motivate them to be involved:

All clinicians want to do the right thing but only have a limited amount of time, so I think you would engage clinicians by showing them the data on...literature...and (explain) that if we do this, women are less likely to end up having this outcome...I think people would be excited for that.

C2

##### Demonstration of Impact

Clinician participants supported the approach of conducting an evidence-based trial before introducing mHealth into practice. Most clinicians were willing to participate in an mHealth trial. The initial steps they suggested included targeting particular groups (eg, women in rural and remote areas) and particular conditions and supporting women from low socioeconomic backgrounds where the prevalence of risk factors is common, and technology can make a difference:

So I think in the right conditions with the right people it would work. I could see that it would certainly work in some of the rural and remote areas...if they could just with an App send in their information and then someone can look at it and just ring them up and reassure them.

C9

## Discussion

### Potential Interests

mHealth technologies for health and lifestyle monitoring have been used in the general population. There is a growing interest in introducing mHealth solutions to support the pregnancy journey, which is the period of a woman’s life that involves significant physiological changes and potential risks [29,44]. In this study, we examined the interests and perceptions of women and clinicians regarding the use of mHealth for health and well-being monitoring during pregnancy.

Our study showed that female participants were open to the use of wearables and health monitoring devices to track health and well-being in general, with most of them having previous experience of using physical activity trackers and mobile apps before and during their pregnancy. This result echoes previous research [25,30], including a study conducted in the Australian context [24]. Despite low interest in monitoring lifestyle behaviors among low-risk pregnant women with no medical problems, all female participants felt comfortable sharing information from wearable and monitoring devices with their clinicians and would have felt motivated if clinicians could review the data. In addition, women with a GDM diagnosis were normally engaged in continuous health monitoring of blood glucose, with data being recorded manually, and would be supportive of an mHealth solution to make the process more efficient.

Clinicians in our study did not use mHealth technology and wearables or prescribe mobile apps in the current practices. However, our findings revealed that there was an overall positive response among these clinicians on the potential benefit of mHealth for monitoring pregnant women’s health and well-being and promoting healthy lifestyle behaviors during pregnancy, similar to the findings of other studies [24]. Despite the concern about women’s anxiety caused by self-interpretation of the data, clinicians were interested in using mHealth monitoring to assist the current practices of risk assessment and regular checks with pregnant women. Our study also highlights that mHealth monitoring is aligned with the trend of extending the context of perinatal care to a longitudinal health and well-being care model [26,42]. It can further serve to empower pregnant women to take more of an active role in their lifestyle behaviors during the antenatal, pregnancy, and postnatal periods.

### Improving Engagement With mHealth Technology

#### Overview

Building a rich and multidimensional data pool is required to identify changes in lifestyle, health indicators, and risk factors associated with pregnancy complications. However, the motivation and sustainability of long-term data collection in pregnant women might be difficult [19,27,30]. We found that different contexts (eg, health status and access to support network) can impact an individual’s decision to track and share data. Our findings have shown that higher compliance can be achieved in women who were already engaged with their care (eg, women with higher risk) and women who embraced technology. Women with busy work commitments were less likely to comply with the use of mHealth solutions.

#### Design Considerations

Our study has shown that monitoring requirements and care needs vary with the combination of particular conditions and risk levels among pregnant women. As such, technology solutions need to be tailored to the unique needs as per the conditions and risk levels of the individual women. Different modules with different monitoring parameters and monitoring frequencies can be made available for clinicians to select and assign to women based on the severity of their conditions and risks. For women considered to be at high risk or for those with an available diagnosis, the focus of the solution can be on using condition-specific devices and parameters to help prevent adverse events and provide alerts to both pregnant women and their clinicians. For low-risk women, to reduce their unnecessary burden and anxiety, the focus of the solution should be to help them establish healthy lifestyle behaviors and watch their symptoms without the daily collection of medical data.

Irrespective of the risk levels, our research suggests that women will benefit from a mobile app that not only interfaces with monitoring devices but also provides guidance on healthy lifestyle and behavior changes. Other studies have shown that mHealth interventions often require support from other modalities, such as educational content [45,46]. Our study has revealed further details about the women’s tendency to seek trustworthy tools that deliver answers to weekly pregnancy and baby growth information, concerns in early pregnancy stage, information support services, and personalized information, such as nutrition, fitness, and weight. Pregnant women require clinically accurate and actionable information and feedback. Simple, engaging, tailored, and risk-appropriate information and text messaging delivered according to their stage of pregnancy can be useful in maintaining pregnant women’s interests and satisfaction. Similarly, motivation tools such as medals and rewards in apps can provide them with encouragement for achievements, such as targeted physical exercises or healthier gestational weight gain.

#### Implementation Considerations

Our study suggests some strategies on how to work best with less motivated pregnant women. First, one possible solution to help overcome this challenge would be to introduce the technology to women during the first trimester, which is a difficult stage when mental health support and self-guided information seeking are needed. This would allow them ample time to get comfortable with the technology and overcome some level of anxiety, thus motivating them for continued use in the later stage. Second, clinicians’ recommendations and indications of potential positive outcomes in women can help improve their acceptance. This may contribute to lesser anxiety and stress and higher motivation and reassurance for the women if they know that clinicians are involved. Finally, providing education and training to women in using technology is also important to reduce unnecessary stress and anxiety associated with mHealth during pregnancy.

### Introducing mHealth Technology in Practices

#### Overview

Given the complexity of pregnancy care, there are challenges in introducing mHealth monitoring in care practices. Detecting clinical abnormalities and analysis based on high volume and heterogeneous data generated from mHealth devices can be challenging. This requires the skills necessary to accurately analyze the data for sound clinical decision-making. Participants of this study were also concerned about the extra workload for clinicians in data monitoring.

#### Design Considerations

Female participants and clinician participants of this study were supportive of having an mHealth system with an alerting function that could not only notify the clinicians of changes in a woman’s condition but also enable the women to be aware of problems and to be proactive in seeking professional service. Research in advancing data mining techniques and personalized algorithms has made intelligent detection and risk awareness possible.

However, according to our interviewed clinicians, accurately predicting the likelihood of a pregnancy risk and change in a condition is difficult in pregnancy care. It might require a multidisciplinary approach that considers pregnancy risk factors, symptoms, laboratory findings, and even data about the baby.

Health monitoring using physiological and activity measures from wearable sensors has been growing recently, but the integration of these technologies into practices, particularly pregnancy care, has been limited due to concerns about patient privacy, uncertainty about the reliability of the technologies, and usability, as reported in other studies [29,30,33,38]. In this study, the women’s views on privacy varied. Some women were not worried about it, whereas others were cautious about providing their data because of concerns regarding the maintenance of confidentiality for the captured data. Uncertainty in the reliability of these emerging wearables was also expressed by the clinician participants. Ease of use and automatic data capture were among the women’s and clinicians’ requirements for the devices. Technology development in truly wearable, miniaturized, and nonintrusive technologies can lower the barrier of usability and allow passive and longitudinal data collection.

#### Implementation Considerations

In this study, the clinician participants anticipated that some medical conditions such as GDM, hypertension, and mental health could benefit from the use of mHealth monitoring. However, evidence on the effectiveness of mHealth monitoring in pregnancy is limited and needs further investigation before supporting its future use. They suggested that some medical conditions (eg, GDM) and groups (women in rural and remote areas) would be suitable for the feasibility trials of mHealth and for further investigations before implementation. Longitudinal studies are needed to evaluate the efficacy of mHealth solutions for monitoring during pregnancy, especially in high-risk pregnancies as well as acceptability among pregnant women and clinicians to promote the uptake of mHealth technology.

### Limitations

Due to the constraints in the hospital ethics application process for studies involving pregnant patients in hospitals, we were not able to recruit female participants from the hospital during the study period. All female participants were recruited through community advertisements and word of mouth. As such, the number of female participants was limited, particularly those in the first trimester of their pregnancy. Additionally, the clinician participants were recruited from a public hospital, although the obstetricians also worked at private hospitals. To enrich the current findings, further studies could gain insights from more clinicians working in private hospitals and the GPs. Finally, in this study, we only captured limited socioeconomic information from female participants. We found that the participants touched upon (only slightly) the challenges for women with low income or women with diverse cultural and linguistic backgrounds during the interviews. Future research on the impact of pregnant women’s socioeconomic status and cultural background might be needed to better understand the technology generalizability and digital equity in mHealth for pregnancy care.

### Conclusions

We have explored the aspects of current risk assessment practices, users’ motivations, and concerns as well as clinical and technical factors that need to be considered when designing and introducing mHealth monitoring solutions for pregnant care. Adequate high-quality data collected through longitudinal monitoring is required for the intelligent detection of risks. We discussed technology solutions and implementation strategies to improve pregnant women’s engagement with technology and data collection, which are critical for mHealth solutions to facilitate the tracking of health and behavior changes during pregnancy. Future research will include feasibility studies to inform the development of mHealth technology and evidence-based evaluation studies to understand the efficacy of mHealth solutions in supporting pregnancy care.

## Abbreviations

GDM: gestational diabetes mellitus
GP: general practitioner
mHealth: mobile health

## References

[1] Kautzky-Willer A, Bancher-Todesca D, Birnbacher R (2004). Gestational diabetes mellitus. Acta Med Austriaca.

[2] Vatten LJ, Skjaerven R (2004). Is pre-eclampsia more than one disease?. BJOG.

[3] Luoto R, Mottola M, Hilakivi-Clarke L (2013). Pregnancy and lifestyle: short- and long-term effects on mother's and her children's health. J Pregnancy.

[4] Oteng-Ntim E, Varma R, Croker H, Poston L, Doyle P (2012). Lifestyle interventions for overweight and obese pregnant women to improve pregnancy outcome: systematic review and meta-analysis. BMC Med.

[5] (2014). National midwifery guidelines for consultation and referral. Australian College of Midwives.

[6] English S, Steele A, Williams A, Blacklay J, Sorinola O, Wernisch L, Grammatopoulos DK (2018). Modelling of psychosocial and lifestyle predictors of peripartum depressive symptoms associated with distinct risk trajectories: a prospective cohort study. Sci Rep.

[7] Abell SK, Shorakae S, Boyle JA, De Courten B, Stepto NK, Teede HJ, Harrison CL (2019). Role of serum biomarkers to optimise a validated clinical risk prediction tool for gestational diabetes. Aust N Z J Obstet Gynaecol.

[8] Moreira MW, Rodrigues JJ, Kumar N, Saleem K, Illin IV (2019). Postpartum depression prediction through pregnancy data analysis for emotion-aware smart systems. Inf Fusion.

[9] Velikova M, Lucas P, Spaanderman M (2011). A predictive Bayesian network model for home management of preeclampsia. Artificial Intelligence in Medicine.

[10] World Health Organization (2011). mHealth: New Horizons for Health Through Mobile Technologies.

[11] Marcolino MS, Oliveira JA, D'Agostino M, Ribeiro AL, Alkmim MB, Novillo-Ortiz D (2018). The impact of mHealth interventions: systematic review of systematic reviews. JMIR Mhealth Uhealth.

[12] Brickwood K, Watson G, O'Brien J, Williams AD (2019). Consumer-based wearable activity trackers increase physical activity participation: systematic review and meta-analysis. JMIR Mhealth Uhealth.

[13] Lu L, Zhang J, Xie Y, Gao F, Xu S, Wu X, Ye Z (2020). Wearable health devices in health care: narrative systematic review. JMIR Mhealth Uhealth.

[14] Boll S, Heuten W, Meyer J (2016). From tracking to personal health. Interactions.

[15] Huang W, Li J, Alem L (2018). Towards preventative healthcare: a review of wearable and mobile applications. Stud Health Technol Inform.

[16] Varnfield M, Karunanithi M, Lee C, Honeyman E, Arnold D, Ding H, Smith C, Walters DL (2014). Smartphone-based home care model improved use of cardiac rehabilitation in postmyocardial infarction patients: results from a randomised controlled trial. Heart.

[17] Shan R, Sarkar S, Martin SS (2019). Digital health technology and mobile devices for the management of diabetes mellitus: state of the art. Diabetologia.

[18] Silvera-Tawil D, Hussain MS, Li J (2020). Emerging technologies for precision health: an insight into sensing technologies for health and wellbeing. Smart Health.

[19] Rivera-Romero O, Olmo A, Muñoz R, Stiefel P, Miranda ML, Beltrán LM (2018). Mobile health solutions for hypertensive disorders in pregnancy: scoping literature review. JMIR Mhealth Uhealth.

[20] Chan KL, Chen M (2019). Effects of social media and mobile health apps on pregnancy care: meta-analysis. JMIR Mhealth Uhealth.

[21] Overdijkink SB, Velu AV, Rosman AN, van Beukering MD, Kok M, Steegers-Theunissen RP (2018). The usability and effectiveness of mobile health technology-based lifestyle and medical intervention apps supporting health care during pregnancy: systematic review. JMIR Mhealth Uhealth.

[22] Payakachat N, Rhoads S, McCoy H, Dajani N, Eswaran H, Lowery C (2020). Using mHealth in postpartum women with pre-eclampsia: lessons learned from a qualitative study. Int J Gynaecol Obstet.

[23] Krishnamurti T, Davis AL, Wong-Parodi G, Fischhoff B, Sadovsky Y, Simhan HN (2017). Development and testing of the MyHealthyPregnancy app: a behavioral decision research-based tool for assessing and communicating pregnancy risk. JMIR Mhealth Uhealth.

[24] Willcox JC, van der Pligt P, Ball K, Wilkinson SA, Lappas M, McCarthy EA, Campbell KJ (2015). Views of women and health professionals on mHealth lifestyle interventions in pregnancy: a qualitative investigation. JMIR Mhealth Uhealth.

[25] Vo V, Auroy L, Sarradon-Eck A (2019). Patients' perceptions of mHealth apps: meta-ethnographic review of qualitative studies. JMIR Mhealth Uhealth.

[26] Shorey S, Yang YY, Dennis C (2018). A mobile health app-based postnatal educational program (Home-but not Alone): descriptive qualitative study. J Med Internet Res.

[27] Chaudhry B, Faust L, Chawla NV (2019). From design to development to evaluation of a pregnancy app for low-income women in a community-based setting. Proceedings of the 21st International Conference on Human-Computer Interaction with Mobile Devices and Services.

[28] Barry M, Doherty K, Marcano BJ, Car J, Morrison C, Doherty G (2017). mHealth for maternal mental healthveryday wisdom in ethical design. Proceedings of the 2017 CHI Conference on Human Factors in Computing Systems.

[29] Penders J, Altini M, Van Hoof C, Dy E (2015). Wearable sensors for healthier pregnancies. Proc IEEE.

[30] Goetz M, Müller M, Matthies LM, Hansen J, Doster A, Szabo A, Pauluschke-Fröhlich J, Abele H, Sohn C, Wallwiener M, Wallwiener S (2017). Perceptions of patient engagement applications during pregnancy: a qualitative assessment of the patient's perspective. JMIR Mhealth Uhealth.

[31] Rodbard D (2016). Continuous glucose monitoring: a review of successes, challenges, and opportunities. Diabetes Technol Ther.

[32] Peake JM, Kerr G, Sullivan JP (2018). A critical review of consumer wearables, mobile applications, and equipment for providing biofeedback, monitoring stress, and sleep in physically active populations. Front Physiol.

[33] Runkle J, Sugg M, Boase D, Galvin SL, C Coulson C (2019). Use of wearable sensors for pregnancy health and environmental monitoring: descriptive findings from the perspective of patients and providers. Digit Health.

[34] Gambhir SS, Ge TJ, Vermesh O, Spitler R (2018). Toward achieving precision health. Sci Transl Med.

[35] Haddad SM, Souza RT, Cecatti JG (2019). Mobile technology in health (mHealth) and antenatal care-searching for apps and available solutions: a systematic review. Int J Med Inform.

[36] Davidson L, Boland M (2021). Towards deep phenotyping pregnancy: a systematic review on artificial intelligence and machine learning methods to improve pregnancy outcomes. Brief Bioinform.

[37] Velikova M, Lucas P, Spaanderman M (2011). e-MomCare: a personalised home-monitoring system for pregnancy disorders. Electronic Healthcare.

[38] Rhoads SJ, Serrano CI, Lynch CE, Ounpraseuth ST, Gauss CH, Payakachat N, Lowery CL, Eswaran H (2017). Exploring implementation of m-Health monitoring in postpartum women with hypertension. Telemed J E Health.

[39] van Kasteren Y, Freyne J, Hussain MS (2018). Total knee replacement and the effect of technology on cocreation for improved outcomes and delivery: qualitative multi-stakeholder study. J Med Internet Res.

[40] Bashi N, Hassanzadeh H, Varnfield M, Wee Y, Walters D, Karunanithi M (2018). Multidisciplinary smartphone-based interventions to empower patients with acute coronary syndromes: qualitative study on health care providers' perspectives. JMIR Cardio.

[41] Cox JL, Holden JM, Sagovsky R (1987). Detection of postnatal depression. Development of the 10-item Edinburgh Postnatal Depression Scale. Br J Psychiatry.

[42] Moore T, Arefadib N, Deery A, West S (2017). The first thousand days: an evidence paper. The First 1000 Days: An Evidence Paper.

[43] Continuous glucose monitoring. Diabetes Australia.

[44] Marko KI, Ganju N, Krapf JM, Gaba ND, Brown JA, Benham JJ, Oh J, Richards LM, Meltzer AC (2019). A mobile prenatal care app to reduce in-person visits: prospective controlled trial. JMIR Mhealth Uhealth.

[45] Rehman H, Kamal AK, Sayani S, Morris PB, Merchant AT, Virani SS (2017). Using mobile health (mHealth) technology in the management of diabetes mellitus, physical inactivity, and smoking. Curr Atheroscler Rep.

[46] Hearn L, Miller M, Fletcher A (2013). Online healthy lifestyle support in the perinatal period: what do women want and do they use it?. Aust J Prim Health.

